# Comprehensive New Insights into Sweet Taste Transmission Mechanisms and Detection Methods

**DOI:** 10.3390/foods14132397

**Published:** 2025-07-07

**Authors:** Yuanwei Sun, Shengmeng Zhang, Tianzheng Bao, Zilin Jiang, Weiwei Huang, Xiaoqi Xu, Yibin Qiu, Peng Lei, Rui Wang, Hong Xu, Sha Li, Qi Zhang

**Affiliations:** 1 College of Food Science and Light Industry, Nanjing Tech University, Nanjing 211816, China; 2 State Key Laboratory of Materials-Oriented Chemical Engineering, Nanjing Tech University, Nanjing 211816, China; 3 Jiangsu National Synergetic Innovation Center for Advanced Materials, Nanjing Tech University, Nanjing 211816, China

**Keywords:** sweetness, transmission mechanism, detection method, sensory evaluation, electronic tongue, biosensor

## Abstract

Sweet taste plays a pivotal role in human dietary behavior and metabolic regulation. With the increasing incidence of metabolic disorders linked to excessive sugar intake, the development and accurate evaluation of new sweeteners have become critical topics in food science and public health. However, the structural diversity of sweeteners and their complex interactions with sweet taste receptors present major challenges for standardized sweetness detection. This review offers a comprehensive and up-to-date overview of sweet taste transmission mechanisms and current detection methods. It outlines the classification and sensory characteristics of both conventional and emerging sweeteners, and explains the multi-level signaling pathway from receptor binding to neural encoding. Key detection techniques, including sensory evaluation, electronic tongues, and biosensors, are systematically compared in terms of their working principles, application scope, and limitations. Special emphasis is placed on advanced biosensing technologies utilizing receptor–ligand interactions and nanomaterials for highly sensitive and specific detection. Furthermore, an intelligent detection framework integrating molecular recognition, multi-source data fusion, and artificial intelligence is proposed. This interdisciplinary approach provides new insights and technical solutions to support precise sweetness evaluation and the future development of healthier food systems.

## 1. Introduction

Sweet taste perception has been an essential biological mechanism throughout human evolution, guiding dietary behavior through the recognition of high-calorie carbohydrates such as glucose and sucrose [1]. This sensory ability supports energy acquisition, contributing to survival and nutritional balance. However, in recent decades, the rapid advancement of agriculture and the food industry has led to excessive consumption of added sugars. This dietary shift is closely associated with the rising prevalence of obesity, type 2 diabetes, and other metabolic disorders, which are now recognized as major public health concerns worldwide [2]. In response, increasing attention has been directed toward the development of low-calorie sweeteners to reduce sugar intake while preserving palatability. Low-calorie sweeteners encompass a wide range of compounds, such as artificial sweeteners and sweet-tasting proteins [3]. While this diversification provides broader options for calorie control, the differences in molecular structure and receptor activation pathways make it difficult to standardize sweetness evaluation across compounds. Furthermore, individual genetic variations and perception mechanisms introduce additional complexity, challenging the establishment of reliable, universal sweetness measurement systems. Hence this study will focus on the comprehensive evaluation of more new sweeteners.

Current sweetness evaluation still largely depends on sensory analysis, including triangle tests and quantitative descriptive analysis [4]. Although international protocols have been established to improve consistency, sensory data often suffer from limited reproducibility due to variations in physiology, cognitive bias, and environmental conditions [5]. To address the limitations of human-based assessment, electronic tongue systems have been developed [6]. These systems use cross-sensitive sensor arrays to simulate taste perception and generate objective “taste fingerprints”. Despite their technical advantages, electronic tongues are limited by their inability to detect non-electroactive compounds and their insensitivity to the interaction between taste and smell, which reduces their applicability in complex food matrices [7]. The identification of the T1R2/T1R3 sweet taste receptor and its associated intracellular signaling pathways has provided a molecular foundation for a new generation of detection technologies [8]. These receptors are heterodimeric proteins embedded in the cell membrane, containing multiple domains capable of binding structurally diverse sweeteners [9]. Based on this molecular understanding, various biosensors have been developed using receptor–ligand interactions, including cell-based platforms expressing T1R2/T1R3 [10] and field-effect transistors functionalized with the venus flytrap domain of T1R2 [11]. These approaches allow for ultra-sensitive detection and the differentiation of sweetener subtypes, shifting the field from subjective evaluation toward objective molecular recognition.

Previous studies have described sweeteners classification and the molecular mechanisms of sweet taste perception [3,12], while this study pays more attention to the logical relationship and close connection between each part, which will clarify the thinking for us to create and invent a new, convenient and sensitive detection system. This study primarily unfolds across three sections: (1) a systematic classification of sweetener types along with their sweetness characteristics; (2) an in-depth analysis of the multi-level signal transduction pathway, from receptor activation to neural encoding, highlighting key biological insights relevant to detection system design; and (3) a comparative evaluation of sensory analysis, electronic tongues, and biosensors, outlining their respective advantages and limitations across various application contexts. Furthermore, this review proposes a forward-looking framework for intelligent, accurate, and scalable sweetness detection. Through an interdisciplinary lens, this work aims to provide new perspectives and practical strategies for the comprehensive evaluation of sweet taste in both scientific research and real-world applications.

## 2. Classification and Characteristics of Sweeteners

**Figure 1:**
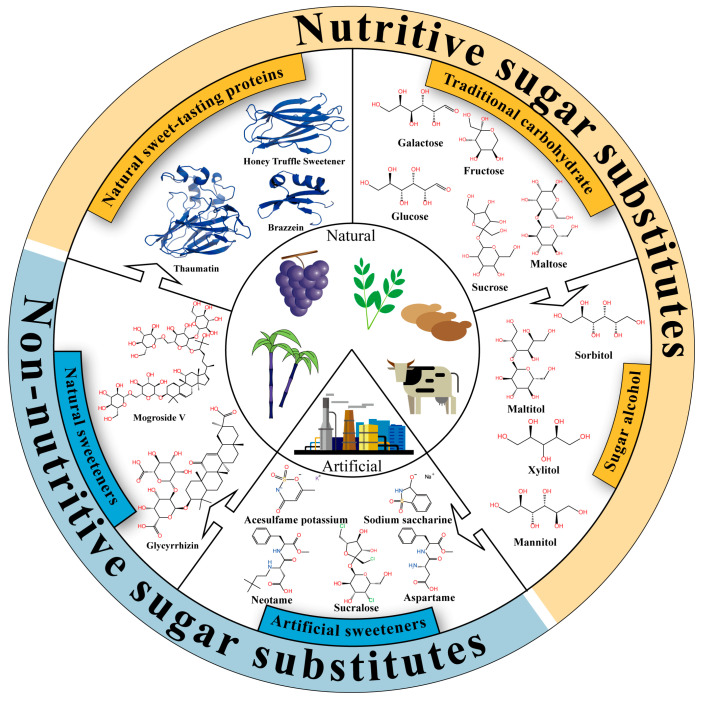
Classification and characteristics of sweeteners. All structural information related to sweet taste proteins was sourced from the RCSB Protein Data Bank (https://www.rcsb.org/).

Sweeteners are classified into five categories based on their structural types and developmental trends: traditional carbohydrates, sugar alcohols, artificial sweeteners, natural non-nutritive sweeteners, and natural nutritive sweet-tasting proteins (Figure 1). Table 1 provides a detailed overview of the classification, characteristics, and sweetness descriptions of these sweet substances. Traditional carbohydrates play a vital role in food formulations. Over the past century, with continuous advancements in breeding, extraction, and purification technologies, the production of traditional carbohydrates has significantly increased, with annual sucrose output exceeding 180 million tons [13,14,15]. The sweetness range of traditional carbohydrates is typically between 0.25 and 1.5, characterized by a mild sweet taste and the absence of off-flavors. As traditional sugars permeate various aspects of modern daily life, researchers worldwide have begun to focus on the health implications of their excessive intake. For instance, high sucrose consumption has been shown to impair children’s memory and learning abilities [16], and sugar-sweetened beverages have been associated with an increased risk of obesity, as well as metabolic syndrome (including insulin resistance, dyslipidemia, elevated blood pressure, and impaired glucose metabolism) [17]. On the other hand, with economic and technological development, the rising incidence of epidemic obesity and diabetes has led to increased public attention to healthy eating. To meet this demand, the food industry has developed sugar alcohol sweeteners, such as xylitol and sorbitol. Although sugar alcohols share structural similarities with traditional sugars, their sweetness and caloric values are generally lower than those of sucrose. Moreover, sugar alcohols serve functions beyond sweetness, including flavor enhancement, coloring, and dental protection. For example, xylitol has been demonstrated to significantly improve oral health in young children by preventing dental caries, supporting enamel remineralization, and inhibiting dental plaque and gingivitis [18].

With the continuous advancement of the food industry, traditional sugars and sugar alcohols can no longer meet consumers’ dual demands for high sweetness and low calories, thus driving the emergence of artificial sweeteners. Artificial sweeteners possess extremely high sweetness intensities, often hundreds of times greater than that of sucrose. For instance, advantame can be 7000 to 47,000 times sweeter than sucrose [19]. Against the backdrop of reduced-sugar diets and rising rates of chronic metabolic diseases, the use of artificial sweeteners partially fulfills the consumer demand for “less sugar without compromising sweetness,” aiding in the control of total caloric intake and blood glucose fluctuations. Additionally, artificial sweeteners offer excellent thermal and storage stability, conferring important technological advantages in food processing sectors such as baking, beverages, and dairy [20]. Currently, sucralose, aspartame, saccharin, neotame, and advantame have all received Generally Recognized As Safe (GRAS) certification from the U.S. FDA. However, despite widespread application, the safety of artificial sweeteners remains controversial. For instance, long-term administration of aspartame exacerbates atherosclerosis in mice [21], cyclamate has been considered potentially carcinogenic or co-carcinogenic [22], and sucralose consumption has been associated with gut microbiota dysbiosis [23]. Furthermore, due to the common belief that “natural is better than artificial,” some consumers show strong psychological resistance toward artificial sweeteners, further propelling the search for more natural and health-promoting alternatives.

Natural sweeteners have gained increasing attention in both academic research and industrial development due to their natural origins, high sweetness, and potential bioactivities. Steviol glycosides and mogrosides are currently the most representative and commercially mature natural sweeteners, while glycyrrhizin has limited applications due to potential adverse effects and lack of GRAS approval. These natural compounds possess various functional properties, including anti-inflammatory [24], antioxidant [24], metabolic regulatory [25], and anti-tumor activities [26]. In practical applications, steviol glycosides often carry a bitter and metallic aftertaste, while mogrosides have a lingering bitter note; therefore, these compounds are typically used in combination with other sweeteners to enhance palatability [27]. In recent years, natural sweet-tasting proteins have emerged as promising high-efficiency sweeteners due to their ultra-high sweetness and low caloric content. Monellin, thaumatin, brazzein, and miraculin are primarily derived from specific plants native to Africa and Southeast Asia [28], while the honey truffle sweetener is extracted from *Mattirolomyces terfezioides* in Hungary [29]. These proteins can be hundreds to thousands of times sweeter than sucrose, showcasing significant potential as sugar substitutes. However, due to the stringent cultivation requirements of source plants and generally low protein yields, natural extraction is insufficient to meet industrial demands. Consequently, current production mainly relies on biosynthetic methods such as microbial fermentation [30]. Moreover, several technical and economic constraints continue to limit their commercial application, including low expression yields, structural instability, poor compatibility with food matrices, and potential allergenicity. To address these challenges, recent research has increasingly focused on genetic engineering, structural modification, and molecular design to enhance thermal stability and flavor compatibility [31,32]. With ongoing advancements in synthetic biology, protein engineering, and food processing technologies, sweet-tasting proteins are poised to become a new generation of functional, sustainable, and highly efficient natural sweeteners.

**Table 1:** Classification, characteristics and sweetness description of sweet substances.

Category	Sweetener	Relative Sweetness	Characteristic	Sweetness Description	References
Traditional carbohydrate	Lactose	0.25	Improves taste, a reducing sugar, low solubility in water	Mild sweetness	[33]
	Maltose	0.33	Enhances flavor and aroma, relatively slow rate of digestion and absorption	Mild sweetness	[34]
	Trehalose	0.45	Improves texture, with good pH and thermal stability, and can be used as a food stabilizer	Mild and pleasant sweetness	[35]
	Galactose	0.6	Essential for human metabolism	Longer-lasting sweetness compared to lactose	[36]
	Glucose	0.7	Highly associated with blood glucose levels and dental caries	Pure sweetness, no adverse sensory characteristics	[37]
	Tagatose	0.92	Low calorie	Pure sweetness, no adverse sensory characteristics	[38]
	Sucrose	1	Serves as the sensory standard for sweetness and enhances flavor	Pure sweetness, no adverse sensory characteristics	[38]
	Fructose	1.5	Relative sweetness decreases as the temperature is raised	Cool sensation with a quick disappearance of sweetness	[39]
Sugar alcohol	Lactitol	0.25–0.4	Possesses good moisture retention, stability, and acid resistance	Mild, refreshing sweetness similar to sucrose, with no aftertaste	[40]
	D-Mannitol	0.5–0.7	Good fluidity and does not affect blood glucose levels	Pleasant sweetness	[41]
	Sorbitol	0.6	Good solubility, high stability, and strong hygroscopicity	Cool and refreshing mouthfeel	[41]
	Erythritol	0.6–0.8	Low hygroscopicity and low water activity	Mild and cooling sweetness	[42]
	Maltitol	0.75–0.9	Does not affect blood glucose levels, has excellent emulsifying stability, significant hygroscopicity, and is non-cariogenic	Pure, smooth and harmonious sweetness; sweetness intensity is proportional to concentration	[43]
	Xylitol	1	Does not significantly affect insulin levels and has dental health benefits	Compared to sucrose, the sweetness begins faster but lasts for a shorter duration.	[44,45]
Artificial sweeteners	Cyclamate	30–50	The recommended maximum daily intake of cyclamate is 11 milligrams per kilogram of body weight. Excessive consumption of cyclamate can severely damage the liver and nervous system.	Good flavor with a slightly sour taste, commonly used in synergy with sodium saccharin	[46]
	Aspartame	160–220	Non-glycemic, excellent stability in solid form, contraindicated in patients with phenylketonuria	Clean sweet taste similar to sucrose, longer aftertaste than sucrose	[47]
	Acesulfame K	200	Acid- and heat-resistant, non-glycemic	Has a slight bitter aftertaste and is often used in combination with aspartame and cyclamate	[48]
	Sodium saccharin	300–450	There are potential risks of genotoxicity and carcinogenicity associated with saccharin. It is non-glycemic.	Bitter or metallic aftertaste	[49]
	Sucralose	400–700	Non-nutritive, zero-calorie, and excellent thermal stability	Pure sweetness similar to sucrose, no adverse sensory characteristics	[48]
	Neotame	6000–10,000	Non-glycemic, excellent stability in solid form	Pure sweetness similar to sucrose, longer aftertaste than sucrose	[47]
	Advantame	7000–47,000	Advantame exhibits higher stability than aspartame, especially under relatively high temperature and pH conditions.	Pure sweetness similar to aspartame, but with a weak bitter and sour aftertaste	[47]
Natural non-nutritive sweeteners	Stevioside	100–350	Low calorie, with good biosafety. Commonly, steviosides include rebaudioside A and rebaudioside M.	Has bitterness and an unpleasant aftertaste, which intensify with increasing concentration; therefore, it needs to be blended with other sweeteners for use.	[38,50]
	Compound Glycyrrhizin	100–500	Low calorie	Sweetness perception is slow after ingestion, with a long-lasting sweetness and a licorice-like aftertaste.	[51]
	Neohesperidin dihydrochalcone	300–500	Low calorie and great stability	Fruit-like sweetness with a refreshing mouthfeel	[50]
	Mogroside	300–563	Low calorie and great stability	Slow onset	[51]
Natural nutritive sweet-tasting proteins	Miraculin/Neoculin	/	Relatively stable within the pH range of 3 to 12 and at temperatures below 100 °C	Possesses taste-modulating functionality, capable of transforming the perception of sourness into sweetness	[52]
	Sweet peptides	6–8	The sweetness intensity of sweet peptides is closely related to the number of sweet-tasting amino acids and the hydrophilicity of the peptide.	The sweetness is relatively mild and may be accompanied by an umami taste.	[53]
	Mabinlin	400	Mabinlin consists of five isoforms, among which Mabinlin II exhibits the highest thermal stability. Its sweetness can be retained for 48 h at 80 °C.	Induces a long-lasting sweetness	[54]
	Pentadin	500	The molecular weight of Pentadin is approximately twice that of its homolog Brazzein, but its sweetness is significantly lower than that of Brazzein	Intensely sweet tasting	[54,55]
	Neoculin/Curculin	550	A homologous dimer composed of two basic subunits, which is unstable and loses activity at temperatures of 50 °C or higher.	A sweet protein with both intense sweetness and flavor-modulating properties.	[52]
	Honey truffle sweetener	2000	Predicted to be non-allergenic, non-toxic, and easily digestible	Apart from its sweetness, no other sensory characteristics have been described	[29]
	Brazzein	2000	Smallest, heat-stable and intensely sweet protein (MW: 6473 Da)	Intense sweetness with no off-flavors and a long-lasting sweet taste	[56]
	Thaumatin	3000	Intensely sweet protein with high thermal stability and pH stability	A slow onset of sweetness followed by a long lingering sweet liquorice-like aftertaste	[57]
	Monelin	3000	Natural monellin consists of two peptide chains of 44 and 50 aa, held together by non-covalent bonds. At low pH and temperatures above 50 °C, monellin becomes inactive and loses its sweetness.	Intense sweetness, but short in duration	[58]

## 3. Mechanisms of Sweet Taste Transduction

### 3.1. Development of Sweetness Models

Since the emergence of humankind, there has been a continuous pursuit of sweeter foods, along with an ongoing exploration of the molecular mechanisms underlying the perception, signal transduction, and pleasurable experience of sweetness. The core of this mechanism lies in why sweet substances with different structural characteristics can all be tasted and exhibit varying degrees of sweetness.

Before the sweet taste receptor was identified, several theories were proposed to explain the molecular mechanisms underlying the sweetness of sweeteners. Among these, the AH-B model, first proposed by R. S. Shallenberger and T. E. Acree in 1967, was one of the earliest and most widely accepted theories [59]. This theory posits that a sweet-tasting molecule must contain both a hydrogen donor (AH) and a hydrogen acceptor (B). The hydrogen donor is typically a hydroxyl or amino group, while the hydrogen acceptor is usually an oxygen or nitrogen atom capable of forming hydrogen bonds. The distance between these two functional groups generally ranges from 2.5 Å to 4 Å [59]. Moreover, sweet taste receptor contains an AH-B structure similar to that of sweeteners. The interaction between the receptor and the sweetener involves hydrogen bond formation.

Since the AH-B model could not explain the sweetness differences between high-intensity sweeteners and regular sweeteners, Kier et al. [60] extended this theory by proposing the AH-B-X sweet taste molecular model. This model introduces a third binding site in addition to the AH-B model. The X site is hydrophobic, electron-rich, and capable of participating in dispersion interactions. This theory accounts for the enhanced sweetness of aspartame compared to traditional sweeteners. Based on the AH-B-X theory, Nofre et al. [61] proposed the Multipoint Attachment (MPA) theory, which postulates that the human sweet taste receptor contains at least eight recognition sites (B, AH, XH, Gl, G2, G3, G4, and D). These eight recognition sites interact with their corresponding sites on sweeteners through ionic bonds, hydrogen bonds, and van der Waals forces. This theory provides a comprehensive explanation of the interaction between the receptor and sweeteners, ranging from highly potent to less effective sweeteners. However, a unified theoretical framework that explains the common structural features underlying the sweet taste of various sweeteners remains elusive.

### 3.2. Sweeteners and Sweet Taste Receptors

**Figure 2:**
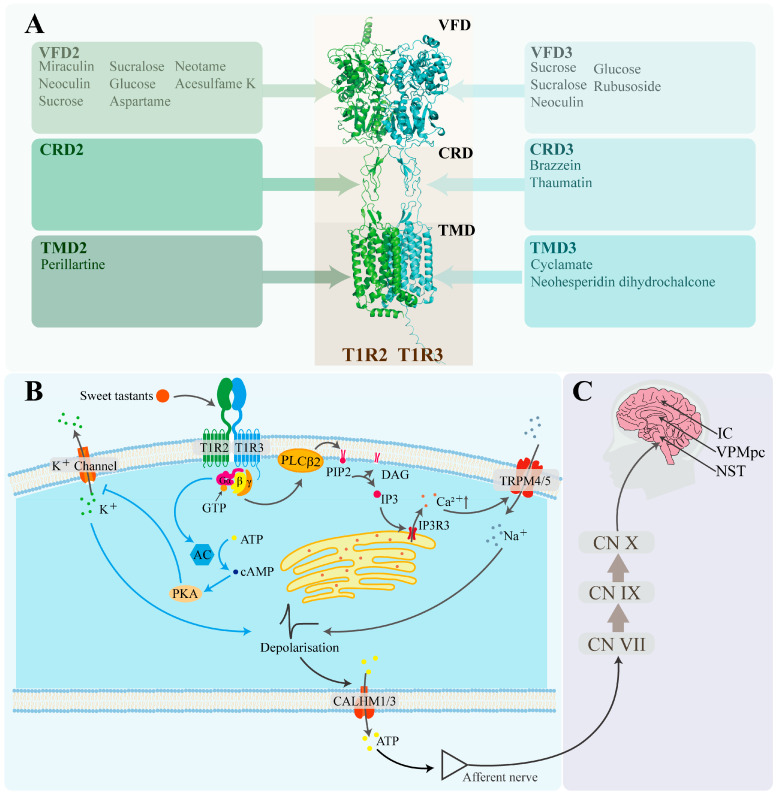
Pathway of sweet taste signal reception, intracellular signaling, and neural conduction. (**A**) Sweeteners and sweet taste receptors (T1R2/T1R3). The structural information was sourced from the RCSB Protein Data Bank (https://www.rcsb.org/). (**B**) Cellular signaling pathways of sweet taste [73]. G_α/β/γ_: guanine nucleotide-binding protein α/β/γ subunits; PLCβ2: phospholipase C β2; PIP2: phosphatidylinositol-4,5-bisphosphate; DAG: diacylglycerol; IP3: inositol 1,4,5-trisphosphate; IP3R3: type III IP3 receptor; Ca^2+^: calcium ion; TRPM4/5: transient receptor potential melastatin channels 4 and 5; Na^+^: sodium ion; AC: adenylate cyclase; GTP: guanosine triphosphate; ATP: adenosine triphosphate; cAMP: cyclic AMP; PKA: protein kinase A; K^+^ channel: potassium ion channel protein; K^+^: potassium ion; CALHM1/3: calcium homeostasis modulator 1 and 3 heterodimer. (**C**) Neural pathways of sweet taste signal transduction [74]. CN VII: facial nerve; CN IX: glossopharyngeal nerve; CN X: vagus nerve; NST: Nucleus of the Solitary Tract; VPMpc: ventroposteromedial nucleus; IC: insular cortex.

The heterodimer T1R2/T1R3 was firstly confirmed to respond to sweet compounds, including sucrose, saccharin, and aspartame, whereas cells lacking either the T1R2 or T1R3 subunit were found to be unresponsive [62]. This finding demonstrates that the heterodimer as a typical member of the G protein-coupled receptor (GPCR) family serves as the primary sweet taste receptor in humans (Figure 2). Despite the identification of the human sweet taste receptor 22 years ago, its three-dimensional structure has yet to be fully resolved. Current studies rely on homology modeling using structurally related templates, such as the Medaka fish sweet taste receptor [63] and the metabotropic glutamate receptors (mGluRs) [64,65,66]. Homologous modeling of the sweet taste receptor has made further discoveries about the mechanism of sweet taste perception and conduction. Each subunit of T1R2/T1R3 is composed of three principal domains: the venus flytrap domain (VFD) located at the extracellular terminus; the seven-transmembrane domain (TMD) located at cell membrane; and the cysteine-rich domain (CRD), which connects VFD with TMD.

Three specific domains of T1R2/T1R3 have been proven to be able to combine with different sweeteners for conducting sweet taste signals (Figure 2A). The structure of VFD contains a hinge-region resembling the leaf of a venus flytrap plant, which is the essential reason enabling VFD to serve as the primary domain responsible for binding sweet-tasting compounds [67] that contain traditional carbohydrates, artificial sweeteners, and natural nutritive sweet-tasting proteins. Glucose and sucrose exhibit binding capacity to the hinge regions of both VFD2 and VFD3. In contrast, taste-modifying proteins exhibit specificity with the binding sites of VFDs. Miraculin binds to VFD2, whereas neoculin interacts with VFD3 [68,69]. Similarly, artificial sweeteners, such as neotame, aspartame and acesulfame K, predominantly bind to VFD2 rather than VFD3 [70]. This may be attributed to the low amino acid sequence homology between VFD2 and VFD3, which is merely 32.91%.

The CRD of T1R2/T1R3 is considered to mainly bind to sweet-tasting proteins. Substitution experiments inserting mouse-derived sweet taste receptor domains into the human sweet taste receptor confirmed that sweet-tasting proteins preferentially bind to the CRD of human T1R3 (CRD3). Peihua Jiang et al. [9] demonstrated that the critical binding site for brazzein is located at residues 536–545 of CRD3. Keisuke Ohta et al. [71] employed the same approach to confirm that the primary binding site for thaumatin also resides within CRD3. Site-directed mutagenesis of T1R3 precisely identified residues 504, 537, 556, 559, and 560 as crucial for thaumatin binding [72].

Certain sweet-tasting compounds can also interact with the TMD domain to transmit a sweet-tasting signal. The TMD domain contains seven typical transmembrane α-helices that are characterized by strong hydrophobicity. Through studies involving chimeric human–mouse receptors and site-directed mutagenesis, the binding sites of saccharin and the sweetness inhibitor lactitol have been identified within the TMD domain of T1R3 [70]. Building upon this, Marcel Winnig et al. further determined the binding site of neohesperidin dihydrochalcone (NHDC) in the TMD domain of T1R3 by analyzing the competitive inhibition effect between NHDC and lactitol, followed by additional site-directed mutagenesis experiments [75].

### 3.3. Cellular Signaling Pathways of Sweet Taste

G proteins involved in sweet taste signaling are heterotrimeric and consist of G_α-gustducin_, G_β3_, and G_γ13_ [76]. Upon recognition of sweet stimuli by G protein-coupled receptors (GPCRs), the receptors undergo conformational changes that activate the intracellular G proteins anchored to the cell membrane, initiating downstream intracellular sweet signaling cascades [77]. Guanosine diphosphate (GDP) bound to the G_α-gustducin_ subunit is exchanged to guanosine triphosphate (GTP). Subsequently, the G_α-gustducin_–GTP complex dissociates from the G_β3_-G_γ13_ dimer, forming a complex signaling network. Depending on the type of secondary messenger involved, sweet taste transduction can proceed through two main signaling pathways—the inositol trisphosphate (IP3) pathway, and the cyclic adenosine monophosphate (cAMP) pathway (Figure 2B)—with the IP3 pathway serving as the primary mechanism for cellular sweet taste perception [78].

In the IP3 pathway, the G_β3_-G_γ13_ dimeric subunit dissociates from the G_α-gustducin_ subunit and can bind to phospholipase C (PLC) β2 [79], thereby activating PLC activity. This catalyzes the hydrolysis of phosphatidylinositol 4,5-bisphosphate (PIP2) on the cell membrane to form IP3 and diacylglycerol (DAG). IP3, as a second messenger, rapidly diffuses after its production and binds to the corresponding receptor (IP3R) on the endoplasmic reticulum or sarcoplasmic reticulum, opening the Ca^2+^ channels (IP3R3) and causing the release of Ca^2+^ into the cytoplasm. This results in an increase in intracellular Ca^2+^ concentration. The elevation of cytosolic Ca^2+^ concentration leads to the opening of the transient receptor potential melastatin channels (TRPM 4 and TRPM5), which are selective for monovalent cations, resulting in an influx of sodium ions and membrane depolarization [80,81,82]. The depolarization then triggers the release of adenosine triphosphate (ATP), a neurotransmitter, through the calcium homeostasis modulator heterodimer (CALHM 1 and CALHM 3), which regulates calcium homeostasis, activating purinergic receptors and initiating taste signal transduction [83,84].

In the cAMP pathway, G_α-gustducin_ binds to GTP and dissociates from the G_β3_-G_γ13_ dimer, which activates adenylate cyclase (AC) to synthesize cAMP [85]. cAMP, as a second messenger, activates cAMP-dependent protein kinase A (PKA). PKA phosphorylates the potassium ion channel protein (K_ATP_) on the cell membrane [86], blocking K^+^ efflux, which increases the intracellular K^+^ concentration and leads to cell depolarization [87,88].

### 3.4. Neural Pathways of Sweet Taste Signal Transduction

The neural transmission pathway of sweet taste signaling in humans can be primarily divided into three stages (Figure 2C). Stage 1: Taste information from the tongue is sequentially transmitted via the facial nerve (cranial nerve VII), glossopharyngeal nerve (cranial nerve IX), and vagus nerve (cranial nerve X), ultimately projecting to the Nucleus of the Solitary Tract (NST) [89]. Stage 2: The NST processes this gustatory information and projects it to the parvicellular portion of the ventroposteromedial nucleus of the thalamus (VPMpc) [90]. Stage 3: Finally, the VPMpc relays the information to the insular cortex (IC). Meanwhile, the IC sends feedback signals to the VPMpc to modulate the processing of gustatory information. Before these signals reach the central neurons through the gustatory pathway, the system encodes taste-related information based on chemical properties, nutritional value, and the intensity of sensory stimulation [74].

## 4. Sweetness Detection Methods

### 4.1. Sensory Evaluation

Sensory evaluation of sweetness primarily relies on gustatory feedback. The assessment of taste characteristics should consider four key aspects: the mode of ingestion, the quantity ingested, the retention time in the mouth, and the specific region of the tongue involved [91]. Based on the purpose of sweetness detection, the target substances, the evaluation content, and the profile of the assessors, sensory evaluation methods can be classified into three categories: discrimination tests, descriptive analyses, and consumer tests.

#### 4.1.1. Discrimination Tests

Discrimination tests are typically used to determine differences in sweetness between two or more samples [92]. Depending on the testing objective, experimental conditions, and methods of data collection, discrimination test methods commonly include the triangle test (TT), two-alternative forced-choice (2-AFC), and three-alternative forced-choice (3-AFC). In the TT method, participants are asked to identify the sample with a perceptible difference in sweetness from a group of three samples, two of which are identical, while the third is different. The 2-AFC method compares a specific attribute (such as sweetness, sourness, or bitterness) between two samples, requiring participants to use sensory perception (typically taste) to determine which sample exhibits a stronger or weaker intensity of the specified attribute. Similarly, the 3-AFC method compares a specific attribute among three samples.

The TT method can be used to conduct comparative taste dilution analysis and determine taste recognition threshold concentrations. It is also an effective method for evaluating participants’ ability to discriminate among samples. For instance, before conducting descriptive sensory analysis of sweet peptides from Puffer fish (*Takifugu obscurus*) muscle, the TT method was employed to determine appropriate dilution levels, ultimately identifying the sweet peptide Tyr-Gly-Gly-Thr-Pro-Pro-Phe-Val [93]. Compared with TT, the 2-AFC and 3-AFC methods provide more targeted and accurate assessments of specific sweetness attributes. However, because the probability of guessing correctly in a 2-AFC test is relatively high, more participants are needed to achieve statistical significance, thereby reducing testing efficiency and increasing labor costs. The 2-AFC method has been used to determine the relative sweetness of natural sweeteners by asking participants to compare a 5% sucrose solution with various test solutions [94]. The process to identify the sweeter sample has been found to be labor-intensive.

Interestingly, Gridgema et al. proposed the “paradox of discriminatory nondiscriminators,” in which participants who were unable to distinguish a different sample during the first-stage triangle test could still perceive differences in the second-stage 3-AFC test [95]. This result is counterintuitive, as the TT method might appear simpler and require only the identification of one different sample, whereas the 3-AFC test demands recognition of attribute differences among three samples. For example, participants were asked to identify the different sample among three by the TT method, and then to select the sweetest sample in a 3-AFC test [5]. Ultimately, the 3-AFC test yielded significantly higher accuracy, indicating superior performance over the triangle test and supporting the paradox of discriminatory nondiscriminators.

#### 4.1.2. Descriptive Analyses

In contrast to discrimination tests, descriptive analyses focus on the detailed description and quantification of the samples’ sensory attributes. This method evaluates not only sweetness, sweetness intensity, and flavor, but also integrates other sensory characteristics such as aroma and texture. Depending on the experimental goals and requirements, descriptive analyses can be categorized into texture and flavor analysis (TFA), quantitative descriptive analysis (QDA), and time–intensity description analysis (TIDA).

TFA involves panelists scoring all texture attributes of a sample (mechanical, geometrical, surface, and moisture-related) and its flavor characteristics (sweetness, duration, off-flavors) comprehensively. QDA allows panelists to independently and accurately quantify the intensity of each sensory attribute that constitutes the overall perception of a sample. Compared to 3-AFC, QDA provides a more holistic assessment. Unlike TFA, QDA is an independent method in which the organizers typically do not participate in evaluation, and the panelists are not required to reach consensus. For example, Laura Ruiz-Aceituno et al. [96] used QDA to independently score each sensory attribute of 12 oligosaccharide sweeteners, including sweet, bitter, cardboard/stale, candyfloss, sour, metallic, salty, crusty bread, perfume flavor, and sweet aftertaste. In contrast to both TFA and QDA, TIDA is a dynamic descriptive sensory method. It offers continuous monitoring of sweetness perception intensity over time, providing a more comprehensive understanding of the temporal evolution of sweetness in foods and beverages. For instance, in the TIDA study comparing older and younger adults’ perception of glucose, significant differences were found in maximum intensity timing and slope, while no significant differences were observed in reaction time or maximum intensity [97].

In addition to using a single sensory evaluation method, combining a discrimination test and descriptive analysis can produce more comprehensive results. Yulu Sun et al. [98] conducted sweetness sensory evaluation of Baijiu (Chinese liquor) using descriptive sensory analysis to score sweetness in 18 Baijiu samples, and then applied 2-AFC testing to distinguish and verify the sweetness characteristics and intensity of potential sweet-tasting compounds. The results not only differentiated the sweetness of various Baijiu samples but also identified three ester compounds as the primary contributors to sweetness. Therefore, integrated sensory evaluation enables both horizontal differentiation of sweetness across samples and thorough characterization of sweetness variations.

Several important considerations must be addressed in descriptive sensory analysis: (1) Odor may alter the intensity and duration of perceived sweetness [99]. For instance, supertasters of propylthiouracil (PROP) experience higher sweetness intensity than non-tasters [100]. (2) The color of test liquid and the presence of non-sweet compounds may influence sweetness perception. Adding citric acid and malic acid, or appropriately coloring the solutions could help participants more accurately identify flavors [101]. (3) Uneven distribution of sweeteners in test samples may cause errors in sweetness evaluation. For instance, without adding any sucrose substitutes in biscuits, uneven distribution of sucrose could increase perceived sweetness by 20% [102]. Therefore, before conducting sweetness sensory evaluations, it is recommended to collect background information on participants and perform homogeneity analysis of test samples to minimize external influences on experimental results.

#### 4.1.3. Consumer Tests

Consumer tests are a special sensory evaluation method that involves having consumers rate or rank products based on various attributes (such as taste, sweetness, bitterness, color, and texture), in order to understand their perception and preferences. Unlike descriptive analysis, consumer tests are typically conducted with untrained individuals from the general public. These consumers tend to make comparative and classification decisions based primarily on the initial few seconds (2–5 s) of sweetness perception experienced in the mouth [103].

The goal of consumer tests is to gather genuine market feedback to assist product developers in determining optimal formulations, refining product characteristics, and predicting market acceptance. For example, in research concerning sucrose replacement in yogurt, most of the 229 recruited consumers preferred natural sweeteners as alternatives, with honey being the most favored option [104]. In another study involving the evaluation of strawberry samples, results from 384 consumers showed that overall liking was closely related not only to sweetness intensity but also to texture and flavor attributes [105].

In summary, consumer testing helps manufacturers better understand market demands, supports product developers in creating new products and improving existing ones, and ultimately enhances product competitiveness. It also increases consumer engagement and loyalty. However, due to individual variability among participants, consumer tests require large sample sizes, which translates into significant investment in manpower and resources.

#### 4.1.4. Advancements in Sensory Evaluation Enabled by Novel Technologies

In recent years, new sensory evaluation indicators and methods have been continuously developed, and emerging technologies have been introduced to improve the accuracy and efficiency of testing in the field of sensory analysis. (1) Biometric Technologies: Techniques such as facial expression analysis, heart rate monitoring, skin conductance, and eye-tracking have been employed to capture the physiological responses of participants during sensory evaluation. These methods provide more comprehensive data on sensory reactions and help reduce subjective bias. For example, the software FaceReader 5.0 can sensitively record subtle changes in facial expressions within 1–3.5 s after the intake of sweeteners [106]. (2) Virtual Reality (VR) and Augmented Reality (AR): These technologies are used to create more immersive and realistic sensory evaluation environments, thereby enhancing the accuracy of evaluation. Previous studies have shown that participants perceive the same beverage as sweeter in a “sweet” environment (with pink/red-shaded colors and soft/smooth visual textures) compared to a bitter environment (black/grey-shaded colors and rough/grainy textures) or a neutral environment (plain white background) [107]. (3) Machine Learning (ML) and Artificial Intelligence (AI): Through deep learning, AI can rapidly analyze large volumes of sensory data to identify correlations, allowing for fast and accurate sweetness assessment in subsequent evaluations. For example, random forest (RF) and classification and regression tree (CART) algorithms have been applied to analyze the relationship between spectral reflectance data (CIE-Lab) and the sweetness intensity of banana, achieving prediction accuracies of up to 86% [108].

### 4.2. Electronic Tongue Technology

The development of objective and efficient intelligent sweetness evaluation technologies is imperative and could serve as an essential supplement to traditional sensory evaluation methods. The electronic tongue, developed over recent decades, is an intelligent taste analysis technology that can simulate the taste assessment process of mammals. It enables rapid identification of the “taste” of liquid samples and provides quantitative technical data on the sensory quality of products. Each type of taste can generate a specific fingerprint signal through the corresponding taste receptor.

#### 4.2.1. Classification and Structure of Electronic Tongues

The electronic tongue consists of a series of non-specific, low-selectivity chemical sensors that exhibit high stability and cross-sensitivity to various substances in solution. The collected data are processed using appropriate pattern recognition and multivariate calibration methods. In 1985, Matthias Otto et al. [109] first proposed using sensors to detect ion concentrations in liquid samples, which is considered the earliest model of the electronic tongue. With continuous technological iterations, modern electronic tongue sensors can be classified based on their working principles into electrochemical sensors, voltammetric/amperometric sensors, impedance sensors, and optical sensors. The main manufacturers in the market are INSENT (Tokyo, Japan), Alpha MOS (Toulouse, France), THINKSENSO&SENSO (Hangzhou, China), and Bosin (Shanghai, China).

Electronic tongues designed for sweetness detection primarily consist of a sensor array, signal acquisition system, and pattern recognition system. The sensor array is the core component of the electronic tongue system. During operation, the array is immersed in a liquid sample, and the membrane on the surface of each sensor electrode reacts with chemical components in the sample, thereby generating electrical signals. These signals are captured and transmitted by the signal acquisition system to a computer, where the pattern recognition system converts them into taste-related information for sample identification and analysis [110]. The sensor array functions analogously to the tongue of mammals, detecting taste-active compounds in the sample, while the pattern recognition system simulates the brain’s decision-making process, similar to the role it plays in electronic nose systems. The mathematical methods used for signal processing are based on pattern recognition and multivariate analysis, such as artificial neural networks (ANN) and principal component analysis (PCA) [111]. The integration of two or more intelligent perception algorithms has become a standard approach in data analysis [112].

#### 4.2.2. Applications of Electronic Tongues in Sweetness Detection

Due to their convenience, accuracy, and efficiency, electronic tongues have been widely employed to detect sweet-tasting components in food and to evaluate quality differences among similar food products, including vegetables (e.g., carrot, soybean), fruits (e.g., sweet orange), tea (e.g., *Moringa oleifera* leaves, *Cyclocarya paliurus* tea), and alcoholic beverages (e.g., Chinese rice wine) [113,114,115]. The core advantage of an electronic tongue in detecting sweetness lies in the fact that it is not influenced by personal emotions, fatigue or subjective preferences, allowing for repeatable and reproducible evaluation of sweetness and other flavor attributes in target liquids. For instance, an electronic tongue can differentiate five soybean varieties based on key characteristic compounds such as isoflavones, sucrose, fructose, glutamic acid, and alanine [115]. An electronic tongue has been applied to determine the sweetness attributes of Chinese rice wine, requiring only 2 min per sample without any need for pretreatment [114]. This demonstrates that electronic tongues offer a short response time and rapid detection speed, enabling fast, non-destructive, and real-time online analysis of the sweetness characteristics of unknown liquid samples. Moreover, electronic tongues can detect all taste thresholds of human sensory perception, including sweetness, sourness, saltiness, umami, bitterness, and astringency. Using a multi-electrode electronic tongue system, Chen et al. [116] identified six key compounds with high sweetness and low bitterness from *Cyclocarya paliurus* tea: cis-anethole, gluconic acid, beta-D-sedoheptulose, asparagine, proline, and citrulline.

#### 4.2.3. Effect of Electronic Tongues on Combined Detection

Although electronic tongues can simulate sweetness perception to a certain extent, they cannot fully replicate all the complex mechanisms of the human sensory system. Previous studies have shown that at least nine fruit aroma compounds can significantly enhance the sweetness of sucrose solutions, with ethyl butyrate being the most prominent [7]. This suggests that fruity aroma compounds may enhance the perception and recognition of sweetness by stimulating both the olfactory and gustatory systems, which an electronic tongue is unable to detect. In binary mixture evaluations, combinations of sweet and bitter compounds do not affect an electronic tongue’s judgment [117], whereas salty and sour substances can interfere with the measurement of sweetness [118]. Therefore, discrepancies may exist between the results obtained from an electronic tongue and human sensory evaluation. From another perspective, combining an electronic tongue with sensory analysis can enable a more comprehensive assessment of sweet-tasting compounds. For example, Yu et al. successfully screened three sweet peptides—Val-Arg-Ser-Tyr, Leu-Tyr-Glu-Arg, and Lys-Gly-Arg-Tyr-Glu-Arg—from Puffer Fish protein hydrolysates using both an electronic tongue and sensory evaluation.

Combining an electronic tongue with various wet-lab identification techniques can further pinpoint sweet components. For instance, using an electronic tongue in conjunction with ultra-performance liquid chromatography coupled with quadrupole time-of-flight mass spectrometry (UPLC-Q-TOF-MS) revealed that the sweet compounds in *Moringa oleifera* leaves may include glucosinolates and flavonoids [113]. Mass spectrometry has been also extensively employed in metabolomic investigations of aroma-active substances in foods such as Peruvian chocolate and black tea [119,120,121]. Moreover, when an electronic nose and electronic tongue were used together, two citrus fruits (*Citrus unshiu* Marc. and *Citrus sinensis*) were perfectly classified with 100% accuracy [122]. Additionally, high correlation coefficients were achieved in models based on a random forest for sensory attributes (training sets > 0.994 and testing sets > 0.983) and volatile components (training sets > 0.992 and testing sets > 0.990), showing an improvement of 8–25% in recognition and prediction capabilities compared to using a single technology alone [7].

### 4.3. Biosensors

With the continued advancement of research on sugar catalysis and heterodimeric human taste receptors, biosensors that rely on artificial lipid membrane sensors have gradually been developed as a powerful complement to electronic tongues. Based on the type of sensing elements employed, mainstream biosensors can be classified into four categories: enzyme-based, non-enzyme-based, taste tissue-based, taste cell-based, and taste receptor-based biosensors.

#### 4.3.1. Enzyme Biosensors

Some sweet-tasting compounds can undergo electron transfer or generate detectable products during enzymatic reactions, which can be converted into measurable signals. By dynamically monitoring the changes in substrate, product, and electron transfer flow using sensors, it is possible to qualitatively and quantitatively detect sweet-tasting substances. Immobilizing specific enzymes on the surface of an electrode enables the construction of electrochemical enzyme biosensors for the qualitative and quantitative detection of sweet compounds.

**Figure 3:**
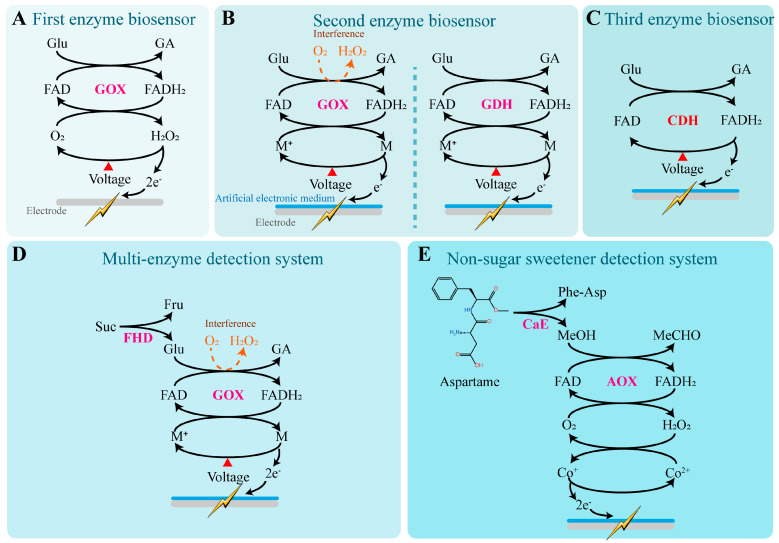
Sweetness detection mechanisms of enzyme biosensors. (**A**) The first type of enzyme biosensor uses H_2_O_2_ as the natural electron donor [123]. In this reaction, glucose oxidase (GOX), which depends on flavin adenine dinucleotide (FAD), catalyzes the oxidation of glucose into gluconic acid (GA), during which FAD is reduced to FADH_2_. Subsequently, GOX (FADH_2_) reacts with molecular oxygen to generate hydrogen peroxide and regenerate the oxidized form of GOX (FAD), thereby completing the catalytic cycle. Under a constant potential, H_2_O_2_ is oxidized at the working electrode surface to produce oxygen while releasing electrons. The resulting electrons generate a weak current on the electrode surface, which can be detected. (**B**) The second type of enzyme biosensor replaces hydrogen peroxide with artificial electron mediators as electron donors [127,128,129]. By using flavin-dependent glucose dehydrogenase (GDH), which is completely oxygen-independent, instead of GOX, interference from oxygen can be entirely eliminated. M: Artificial electronic mediator. (**C**) The third type of enzyme biosensor uses modified enzymes directly as electron carriers [130]. CDH: Cellobiose dehydrogenase. (**D**) Multi-enzyme systems are commonly used for the quantitative detection of oligosaccharides [131]. FHD: Fructoside hydrolase. (**E**) Enzyme biosensors for detecting the content of artificial sweeteners [132]. CaE: Carboxyl esterase.

Based on the type of electron donor used, enzyme biosensors can be categorized into three types (Figure 3). The first type of enzyme biosensor uses H_2_O_2_ as the natural electron donor and was originally developed to determine glucose levels in plasma [123] (Figure 3A). In this type, glucose oxidase (GOX), which is dependent on flavin adenine dinucleotide (FAD), catalyzes the oxidation of glucose to gluconic acid (GA), during which FAD is reduced to FADH_2_. Subsequently, GOX (FADH_2_) reacts with molecular oxygen to produce hydrogen peroxide (H_2_O_2_), regenerating oxidized GOX (FAD) and completing the catalytic cycle [124]. Quantitative detection of sweet substances using this method can be achieved by measuring either the consumption of dissolved oxygen or the generation of gluconic acid or hydrogen peroxide. Continuous synthesis of gluconic acid leads to a decrease in the system’s pH, enabling indirect quantification of glucose concentration by monitoring changes in plasma pH [123]. Under constant potential conditions, H_2_O_2_ is oxidized at the working electrode surface to form oxygen and protons, releasing electrons. The resulting electron flow generates a weak current at the electrode surface, and the current intensity is positively correlated with the H_2_O_2_ concentration, which in turn, indirectly reflects the glucose concentration in the solution [125,126].

The second type of enzyme biosensor incorporates artificial electron mediators into the electrode materials, such as ferrocene [129], ferricyanide [128], and 1T-phase transition metal dichalcogenides [127]. These diffusible mediators can shuttle electrons between the electrode surface and GOX (FADH_2_), effectively replacing H_2_O_2_ as the electron doner. This approach addresses the major limitation of the first-generation biosensors, which heavily rely on dissolved oxygen. Additionally, the introduction of materials like graphene oxide and multi-walled carbon nanotubes enhances the electrode’s conductivity and enzyme-loading capacity, enabling rapid response to glucose concentrations as low as 0.2–5 mM [133]. To further eliminate oxygen interference, researchers have proposed replacing GOX with flavin-dependent glucose dehydrogenase (GDH), an enzyme that operates completely independently of oxygen. This modification allows for a reliable linear response to glucose concentrations ranging from 0 to 20 mM [134]. By substituting enzymes with different substrate specificities or constructing multi-enzyme systems, this type of biosensor can also detect a wider array of sweet-tasting compounds such as fructose [135], lactose [136], and sucrose [137]. These sensors have been widely applied in portable glucose meters, as well as in food and fermentation process monitoring.

The third type of enzyme biosensor builds upon the second-generation system by simplifying the configuration (eliminating the artificial electron mediators) and utilizing engineered enzymes that can directly transfer electrons to the electrode, ensuring safer and more stable electron conduction. This design avoids signal instability and potential toxicity caused by mediator diffusion or degradation. Since electron tunneling distances are typically limited to approximately 1.4 nm, this mechanism requires the enzyme’s active site to be positioned in close proximity to the electrode surface to achieve efficient electron coupling. For example, cellobiose dehydrogenase (CDH) [130], fructose dehydrogenase (FDH) [131], and pyranose dehydrogenase (PDH) [138] have been demonstrated to enable direct electrochemical detection of glucose, fructose, and aldose sugars, respectively, when immobilized on conductive platforms such as carbon nanotubes, gold electrodes, or graphene. These sensors exhibit excellent structural flexibility and biocompatibility, indicating strong potential for applications in wearable devices and real-time monitoring of body fluids.

Multi-enzyme systems are commonly used for the quantitative detection of oligosaccharides. Fructoside hydrolase and glucose oxidase (GOX) have been co-immobilized on gold wire screen-printed electrodes, enabling the detection of sucrose concentrations in tea and juice [139]. By selecting enzymes with broader substrate specificity, monospecific enzyme systems can also detect disaccharides. For example, FAD-dependent glucose dehydrogenase derived from *Trichoderma viride* has been used independently to quantify maltose concentrations, and similarly, maltotriose can also be quantitatively measured [140].

Due to their structural differences from sugars, artificial sweeteners typically require the development of specialized enzyme systems. For instance, to detect the concentration of aspartame, a dual-enzyme system was constructed by immobilizing carboxyl esterase (CaE) and alcohol oxidase (AOX) on screen-printed electrodes modified with cobalt-phthalocyanine (CoPC) [132]. CaE hydrolyzes aspartame into methanol (MeOH), phenylalanine, and aspartyl dipeptide. AOX then oxidizes methanol into formaldehyde (MeCHO) with the concurrent production of hydrogen peroxide (H_2_O_2_). The H_2_O_2_ reduces Co^2+^ on the sensor surface to Co^+^, which transfers electrons to the electrode, thereby achieving the biosensing of aspartame through a dual-enzyme system.

Enzyme-based biosensors demonstrate excellent qualitative and quantitative capabilities for the online detection of specific sweeteners, particularly traditional carbohydrates. They offer advantages such as short detection times and extended operational lifespans. However, to assess the overall sweetness characteristics of a sample, additional types of biosensors may still be required for complementary analysis.

#### 4.3.2. Non-Enzymatic Biosensors

Enzyme-based biosensors are highly susceptible to environmental factors, which can compromise their stability and accuracy. This has sparked growing interest in non-enzymatic sensors, particularly in the fields of clinical diagnostics, the food industry, and biotechnology [141]. A variety of materials—such as graphene, noble metals, transition metal oxides, and composite materials—have been investigated for their potential to enhance the development and performance of non-enzymatic glucose sensors [142,143], offering promising alternatives to enzyme-based biosystems across a broad spectrum of applications.

Non-enzymatic glucose monitoring based on nanostructured materials has garnered unprecedented attention from researchers due to their effective and facile catalytic properties [142]. Among them, Au nanoparticles–Prussian blue analogue nanocomposites have demonstrated low oxidation potentials and excellent electrocatalytic performance for glucose detection [144]. These materials significantly enhance electron transfer rates and exhibit good reusability, showing great potential for low-cost, high-throughput glucose sensing. Wang et al. synthesized a CoMn_2_O_4_@Ni(OH)_2_ nanocage composite using the Kirkendall effect [145]. The stable three-dimensional hollow structure of CoMn_2_O_4_ nanocages provides an enlarged specific surface area and abundant active sites. Moreover, it facilitates ion diffusion, shortens electron transfer pathways, and improves electrical conductivity. This non-enzymatic biosensor exhibited outstanding performance, including a wide linear detection range (8.5–1830.5 μM), low detection limit (0.264 μM), and high sensitivity (0.00646 μA mM^−1^ cm^−2^), enabling sensitive and rapid detection of trace glucose. In addition, a non-enzymatic glucose sensor based on phenylboronic acid-functionalized conductive polymer Kousseff (PEDOT-PBA) integrates both conductive and receptor components within a single organic matrix, effectively addressing the interfacial complexity common in composite materials [146]. As a result, it achieves a lower detection limit, a reduced standard deviation, and a broader linear output range in glucose sensing.

With the development and application of advanced materials, glucose monitoring has gradually evolved from invasive techniques to wearable devices [147]. Increasing attention has been directed toward the possibility of using body fluids—such as blood, sweat, tears, and saliva—for non-invasive or minimally invasive monitoring through skin-attached or subcutaneous devices [148]. These non-enzymatic biosensors, based on materials including carbon-based nanostructures, metal nanoparticles, polymers, and hydrogel systems, are primarily categorized into electrochemical and optical glucose sensing platforms [149]. These wearable systems exhibit high flexibility and stretchability, allowing them to conform to body movements during daily physical activities [149]. However, most current wearable glucose nanoprobes are still limited by high production costs, low biocompatibility, and time-consuming fabrication processes [147]. Therefore, the development of nanomaterials with rapid response times, broad detection ranges, low detection limits, and high selectivity remains a critical challenge.

#### 4.3.3. Taste Bud Tissue Biosensors

**Figure 4:**
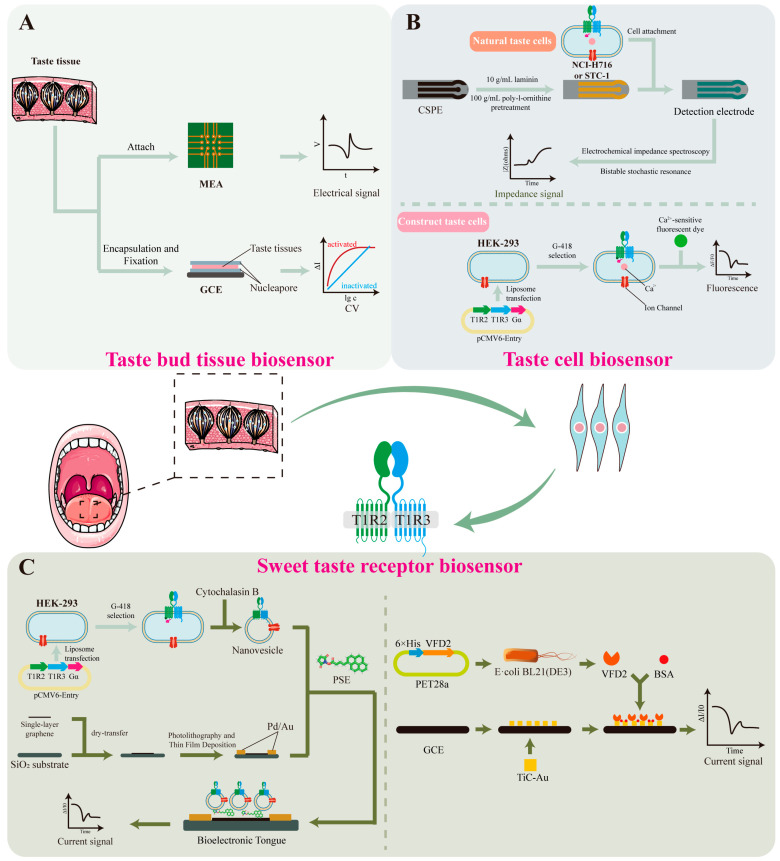
Sweetness detection mechanisms based on taste bud tissue, taste cells and sweetness receptor biosensors. (**A**) Taste bud tissue biosensor [152,153]. Microelectrode arrays (MEA) or glassy carbon electrodes (GCE) have been used to record the electrophysiological signals of taste epithelial tissue. (**B**) Taste cell biosensor [154,155]. Native taste cells can be immobilized on the surface of carbon screen-printed electrodes (CSPE) using poly-L-ornithine or laminin. Signal analysis is conducted using electrochemical impedance spectroscopy and bistable stochastic resonance techniques. Conventional cell lines can be constructed to taste cells by introducing sweet taste receptor (T1R2/T1R3) and Gα genes. Upon sweetness stimulation, the intracellular Ca^2+^ concentration can be monitored using calcium-sensitive fluorescent dyes such as Fluo-3. (**C**) Sweetness receptor biosensor [67,156]. Nanovesicles containing intact taste receptors and ion channels can be obtained by treatment with the cytoskeletal disruptor cytochalasin B. These nanovesicles are then functionalized by immobilizing them onto a single-walled carbon nanotube field-effect transistor surface using 1-pyrenebutyric acid N-hydroxysuccinimide ester (PSE). VFD2 domain of T1R2 can be covalently linked via cysteine residues to the surface of a carbon nanotube field-effect transistor with a floating electrode. Bovine serum albumin (BSA) is used as adjuvant.

Taste bud tissue, as a primary gustatory receptor, can effectively preserve the inherent receptor cell populations, sensory units, and their biological microenvironment (Figure 4A). Therefore, it is considered a highly efficient sensory element for sweetness detection [150]. By integrating taste epithelial tissue with sensors, real-time monitoring of gustation-related electrical or physiological signals can be achieved. To obtain intact taste epithelial tissue, enzymatic digestion can be performed by injecting a mixture containing collagenase A, dispase II, and trypsin inhibitor beneath the lingual epithelium of mice, thereby enabling successful isolation of complete taste epithelium [151]. The isolated tissue can then be immobilized on the surface of an electrochemical sensor for taste signal measurement.

Microelectrode arrays (MEA) or glassy carbon electrodes (GCE) have been used to record the electrophysiological signals of taste epithelial tissue. Wu et al. [153] placed intact taste bud tissue slices onto MEA chips and used the chip to measure the electrophysiological responses of the taste epithelium to glucose, sucrose, saccharin, and cyclamate. Fan et al. [152] immobilized taste bud tissue on the surface of a GCE using starch and sodium alginate as fixing agents to form a “sandwich-structured” membrane, and recorded the taste signals using a three-electrode system. Response currents were measured using cyclic voltammetry (CV) and current–time (I–t) techniques.

Both methods could effectively detect and analyze the electrophysiological responses of taste epithelium to different sweeteners, revealing the spatiotemporal characteristics of sweet-tasting compounds and enabling sweetness evaluation. However, since taste epithelial tissue is derived from living animals, the acquisition of experimental material is complex, preservation is difficult, and the viability of taste tissue is limited after excision. Moreover, due to interspecies differences, the taste receptors of laboratory animals such as mice may respond differently to those of humans to certain sweeteners [9]. Therefore, biosensors based on taste tissue still face considerable challenges in practical applications.

#### 4.3.4. Taste Cell Biosensors

Taste cells have been found not only in taste bud tissues, but are also widely distributed in other tissues such as the myocardium and gastrointestinal tract. For instance, the NCI-H716 [157] and STC-1 cell lines [154] contain receptors capable of detecting sweet, sour, salty, and bitter stimuli, as well as the associated signal transduction pathways, making them directly applicable for constructing taste sensors (Figure 4B). Previous studies have shown that these cells can be immobilized on the surface of carbon screen-printed electrodes (CSPE), which are pre-coated with 100 μg/mL poly-L-ornithine and 10 μg/mL laminin to enhance cell adhesion. Cell lines that do not express taste receptors are used as reference controls to verify system specificity. Signal analysis is conducted using electrochemical impedance spectroscopy and bistable stochastic resonance techniques. Results demonstrated that when NCI-H716 cells are used as the biosensor, they can quantitatively detect seven different concentrations of sucrose solution [154]. When using STC-1 cells, the sensor can not only distinguish sweetness but also exhibits higher sensitivity to bitter compounds, thereby expanding the applicability of the system [154].

Compared to native taste cells, conventional cell lines offer advantages such as lower culture costs, easier manipulation, and higher accuracy. Therefore, researchers have introduced sweet taste receptor genes (T1R2/T1R3) into cell lines lacking endogenous taste receptors (e.g., HEK293), and co-expressed the Gα protein to participate in sweet taste signal transduction. This endowed the engineered cells with sweet-taste sensing capability through the IP_3_ signaling pathway, enabling the construction of a functional sweet-taste sensing cell model. Upon sweet stimulation, the intracellular calcium ion concentration in these cells increases significantly. This change can be monitored using calcium-sensitive fluorescent dyes such as Fluo-3 to indicate variations in Ca^2+^ levels. This method is applicable not only to traditional sweeteners like sucrose but also exhibits good performance in detecting artificial sweeteners and natural nutritive sweet-tasting proteins [155], such as neotame, aspartame, and thaumatin. The calcium-sensitive biosensor GCaMP6s can serve as a substitute for calcium-sensitive fluorescent dyes, simplifying the detection process and improving efficiency. Using this system, the sweetness levels of sucralose, neotame, acesulfame K, perillartine, and sodium cyclamate have been effectively evaluated [10].

Taste cell-based biosensors feature broad detection targets and high sensitivity, making them suitable for high-throughput screening. However, the detection process requires sophisticated equipment and technical expertise, and taste cells show limited tolerance under the harsh conditions of industrial testing, which restricts their practical applications to some extent.

#### 4.3.5. Sweet Taste Receptor Biosensors

Biosensors based on sweet taste receptors have emerged as a more promising option due to their low environmental requirements, minimal individual variability, and long operational lifespan (Figure 4C). Using engineered sweet taste-sensing HEK293 cells, nanovesicles containing intact taste receptors and ion channels can be obtained by treatment with the cytoskeletal disruptor cytochalasin B. These nanovesicles are then functionalized by immobilizing them onto a single-walled carbon nanotube field-effect transistor (SWCNT-FET) surface using 1-pyrenebutanoic acid N-hydroxysuccinimidyl ester (PSE). The resulting biosensor enables highly sensitive and selective detection of both traditional carbohydrates (e.g., sucrose and fructose) and artificial sweeteners (e.g., aspartame and saccharin) [158]. By co-expressing human umami receptors T1R1/T1R3 and sweet taste receptors T1R2/T1R3 on nanovesicles, simultaneous detection of umami and sweet stimuli can also be achieved [156].

As demonstrated in Section 3.2, different domains of the T1R2/T1R3 receptor complex exhibit specific binding affinities toward various sweeteners. Therefore, characteristic domains can be isolated and used as selective sensing elements. For instance, the VFD2 domain of T1R2 is capable of binding to sweeteners such as sucrose, glucose, and fructose [159]. Upon sweetener binding, the VFD2 domain undergoes allosteric conformational changes, altering the surface charge distribution and modulating the Schottky barrier height. Based on this strategy, the VFD2 domain was covalently linked via cysteine residues to the surface of a carbon nanotube field-effect transistor with a floating electrode. Binding of sweeteners to VFD2 led to detectable changes in channel current. Studies have shown that this system can detect sucrose at concentrations as low as 0.1 fM—approximately 10^7^ times more sensitive than previously reported methods [67]. Substituting cysteine with TiC-Au as the anchoring agent further improved the linear response range for both sucrose and aspartame. In stability tests, the sensor exhibited only a 14.3% signal decline over the first five days, demonstrating excellent specificity, concentration-dependence, and durability, and suggesting its strong potential for industrial sweetener detection [11].

These findings inspire the development of sweetener-specific detection kits based on various structural domains of sweet taste receptors, which could shape future research directions in sweet taste receptor-based biosensors. A similar approach has already been applied to umami taste receptor biosensors, using selected domains to specifically detect monosodium glutamate and inosinate, with linear responses to varying concentrations in natural umami samples [160,161].

## 5. Challenges and Perspectives

From the perspective of the molecular level, although the multi-domain synergy of the sweet taste receptor T1R2/T1R3 has been extensively studied, its full three-dimensional structure remains unresolved [162]. This greatly limits the structure-based design and optimization of sweet-tasting molecules. The accurate localization of receptor binding sites still relies on homology modeling, which is limited in precision and unable to fully reveal the underlying mechanisms of action. Furthermore, the intracellular IP3 and cAMP signaling pathways involved in sweet taste transduction interact in complex ways. The cross-talk between these pathways and their regulation of downstream effectors remain to be fully elucidated, making it difficult to faithfully replicate the real taste perception process in biosensors.

From the perspective of sweet taste detection technology applications, electronic tongue systems can rapidly generate “taste fingerprints” and eliminate subjective bias. However, they fail to respond effectively to non-electroactive molecules and are limited in capturing the synergistic sweetness-enhancing effects between taste and olfaction, reducing their accuracy in complex food matrices [7]. Biosensors have achieved breakthroughs in molecular specificity and ultra-high sensitivity. Technologies based on cells or receptor chips can recognize various sweeteners with extremely low detection limits. However, their preparation involves complex steps such as receptor protein expression, purification, and directional immobilization, resulting in high costs. Moreover, signal drift and receptor deactivation during long-term use remain unresolved, directly affecting sensor stability and reproducibility [150]. In addition, the integration of sensory evaluation, electrochemical signals, and molecular recognition results through multimodal data fusion has been proposed as a core concept for intelligent detection systems. However, mature solutions are still lacking in areas such as data standardization, algorithm optimization, and the transition from laboratory research to industrial-scale application.

In future, more efforts should focus on the following directions: (1) Employing high-resolution cryo-electron microscopy combined with molecular dynamics simulations to fully resolve the three-dimensional conformations of the T1R2/T1R3 domains and identify their key binding sites to provide a solid foundation for molecular design and receptor engineering. (2) Applying deep learning and multivariate statistical methods to perform real-time intelligent analysis of multi-source data, to construct integrated models encompassing sensory evaluation, electronic tongue output, and receptor responses, in order to improve prediction accuracy and enable online monitoring. (3) Integrating nanomaterials with microfluidic technologies to design portable and modular detection chips, enabling rapid and low-cost sweetness assessment in on-site detection or consumer-end scenarios. (4) Promoting the standardization of detection methods and results across the industry by establishing unified calibration and validation procedures to ensure data comparability and reliability across laboratories and industrial settings. Through the deep integration of biology, materials science, and information science, the goal is to ultimately achieve precise, intelligent, and efficient full-chain sweetness evaluation—from receptor recognition to holistic perception.

## Data Availability

No new data were created or analyzed in this study. Data sharing is not applicable to this article.
